# Metachronous primary colon and periampullary duodenal cancer: A case report

**DOI:** 10.1097/MD.0000000000024378

**Published:** 2021-01-22

**Authors:** Tao Li, Xinyu Wang, Chen Chen, Xiaobin Song, Jiannan Li, Zeyun Zhao, Nan Zhang, Wei Li, Kai Zhang, Tongjun Liu

**Affiliations:** a Department of Colorectal and Anal Surgery, the Second Hospital of Jilin University, Changchun; b Department of Emergency Surgery, Jilin Province People's Hospital, Changchun; c Department of Burn, the First Hospital of Jilin University, Changchun, Jilin Province, China.

**Keywords:** colon cancer, metachronous double primary malignancies, multiple primary malignancies, periampullary duodenal cancer, tumor sites

## Abstract

**Rationale:**

Primary periampullary duodenal cancer accounts for 3% to 17% of periampullary cancers. There are no previous reports of metachronous primary colon and periampullary duodenal cancer.

**Patient concerns:**

We present a case of primary periampullary duodenal cancer that occurred metachronously after colon cancer.

**Diagnoses:**

Imaging and endoscopic examinations, serum tumor marker levels, and pathology confirmed metachronous colon and periampullary duodenal cancer, with 14-month interval between the diagnoses of the 2 malignancies.

**Intervention:**

The patient received right hemicolectomy combined with mFOLFOX6 chemotherapy for colon cancer and pancreatoduodenectomy for periampullary duodenal cancer.

**Outcomes:**

The patient has been followed up for 6 years since the pancreatoduodenectomy and shows no signs of recurrence or metastasis.

**Lessons:**

The risk of developing a second malignancy may be associated with the site of the first tumor. Patients with right colon cancer may have particularly high risk of developing small intestinal cancer, including duodenal cancer. Early detection and active surgical treatments can improve prognosis. Long-term regular follow-up is necessary to detect new malignancies occurring after the diagnosis colon cancer.

## 1 Introduction

Periampullary duodenal carcinoma is a rare malignancy that arises from the distal common bile duct, pancreatic duct, or ampulla of Vater and the adjacent duodenum.^[1–4]^ It accounts for 3% to 17% of all periampullary cancers.^[5]^ We report a patient who developed a periampullary duodenal cancer 14 months after diagnosis of the colon cancer. This case report highlights the importance of long-term follow-up and regular examinations to detect new malignancies after treatment of colorectal cancer.

## 2 Case presentation

**Figure 1:**
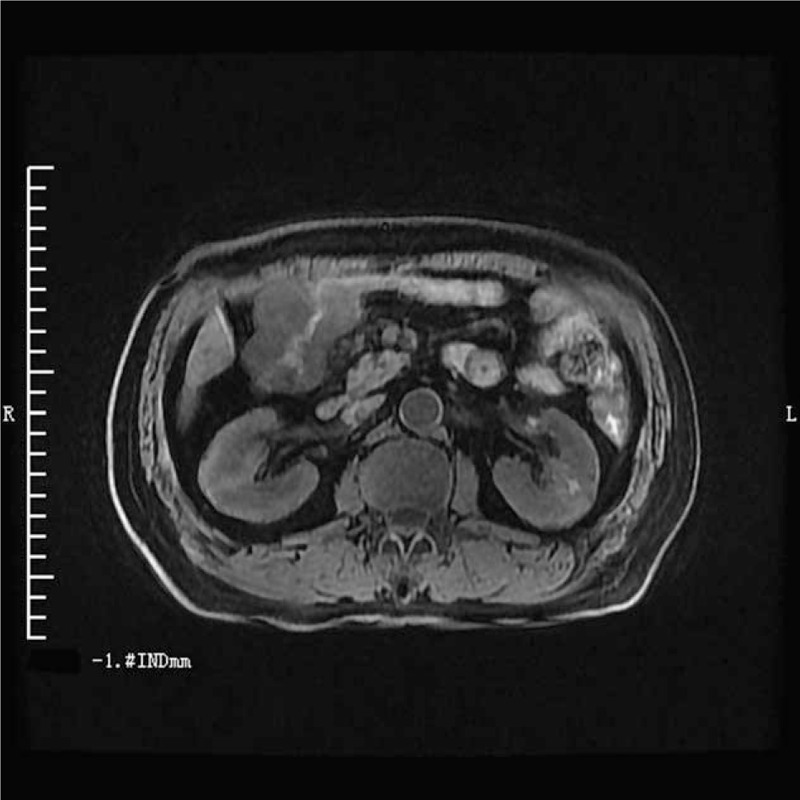
Imaging findings. Abdominal MRI showed an irregular thickening of the hepatic flexure of colon with narrow enteric cavity.

**Figure 2:**
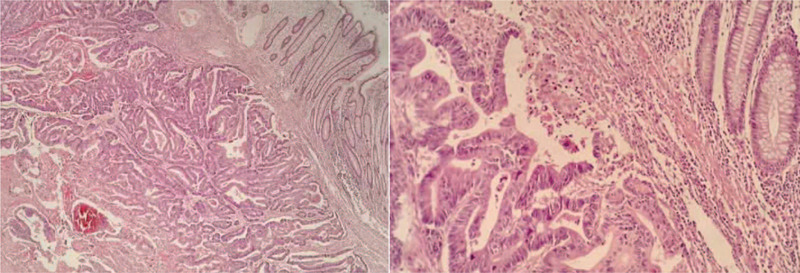
Histopathological findings. Postoperative pathology revealed a moderately to highly differentiated adenocarcinoma. (hematoxylin and eosin staining; 40 × and 200 ×).

A 69-year-old man visited a local hospital complaining of abdominal discomfort and distension. On examination he was found to have a lump in the right upper abdomen. Ultrasound examination showed thickening of the hepatic flexure of the colon and the pseudokidney sign. The richly vascularized thickening involved about 8.3 cm of the colon, with the maximum thickness being about 2.4 cm. The patient was referred to our hospital along with the ultrasound report. He reported that his abdominal distension had been relieved after he had passed stools. Tumor marker levels were elevated: carcinoembryonic antigen (CEA) was 61.42 ng/mL (0–5 ng/mL), carbohydrate antigen 19-9 (CA 19-9) was 483.11 kU/L (0-35 kU/L), and carbohydrate antigen 242 (CA242) was 165.37 kU/L (0–20 kU/L). Magnetic resonance imaging showed irregular thickening of the colon wall at the hepatic flexure, with narrowing of the lumen (Fig. 1). Colonoscopy revealed a wide-based 0.4 cm × 0.4 cm smooth-surfaced cauliflower-like polyp almost filling the lumen. Pathologic examination of the biopsy specimen showed moderately well-differentiated adenocarcinoma. Right hemicolectomy was performed. Postoperative pathology confirmed a moderately to highly differentiated adenocarcinoma, with invasion of the serosa, a tumor thrombus in the vessel, and a carcinoma nodule (Fig. 2). The surgical margin was negative, and there was no lymph node metastasis (0/15). TNM staging was T_3_N_0_M_0_.

Chemotherapy (mFOLFOX6) was started on postoperative day 16. The patient was discharged after the first chemotherapy session. He returned for 2 further chemotherapy sessions, but then refused further treatment. However, he continued to come for follow-up. At 1 month after discharge, the tumor marker levels were as follows: CEA, 4.97 ng/mL; CA 19-9, 300.64 kU/L; and CA242, 100.41 kU/L. Follow-up abdominal and pelvic computed tomography (CT) and colonoscopy performed at 8 months after discharge from hospital showed no evidence of recurrence or metastasis.

**Figure 3:**
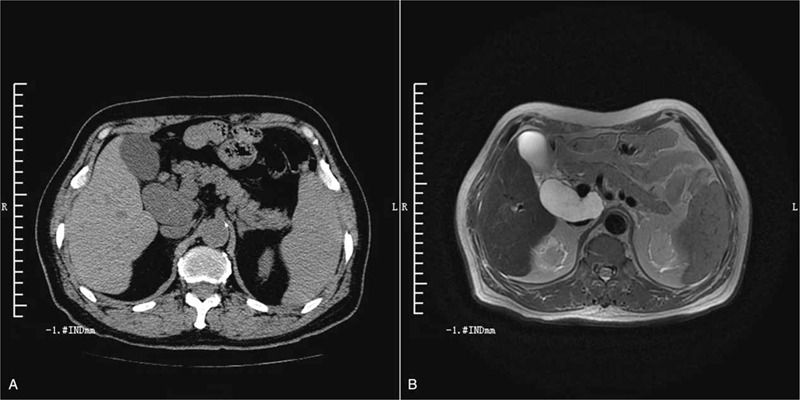
Imaging findings. A. Abdominal CT scan showed a cystic mass behind the head of pancreas. B. Abdominal MRI showed the mass surrounding the portal vein.

**Figure 4:**
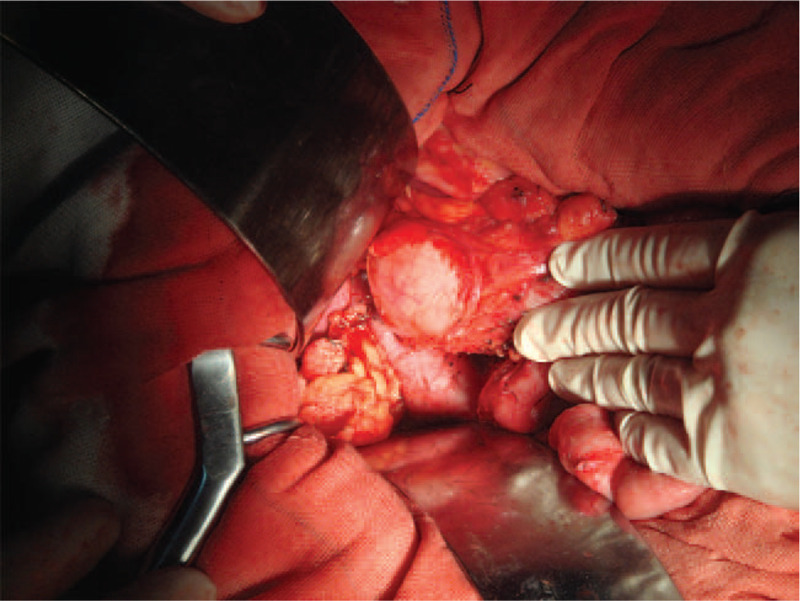
Intraoperative photograph shows the cystic tumor.

**Figure 5:**
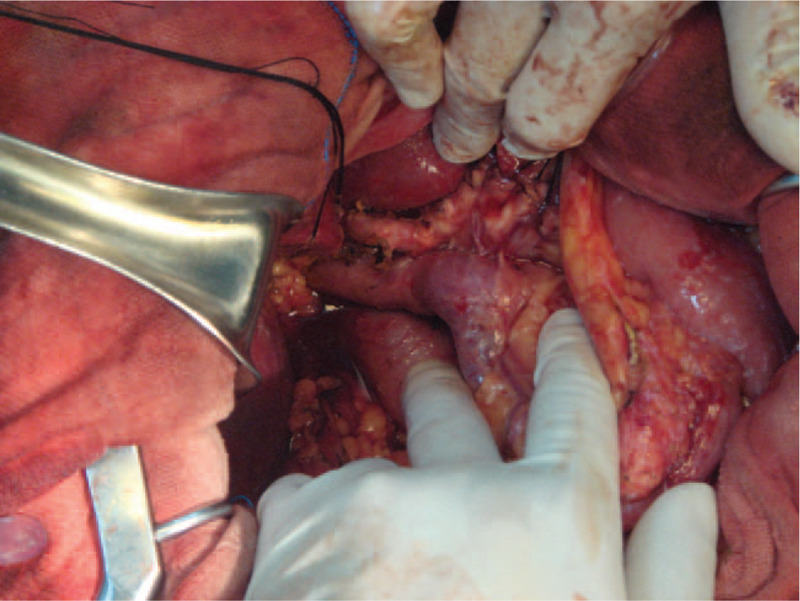
Intraoperative photograph taken after removal of the tumor.

**Figure 6:**
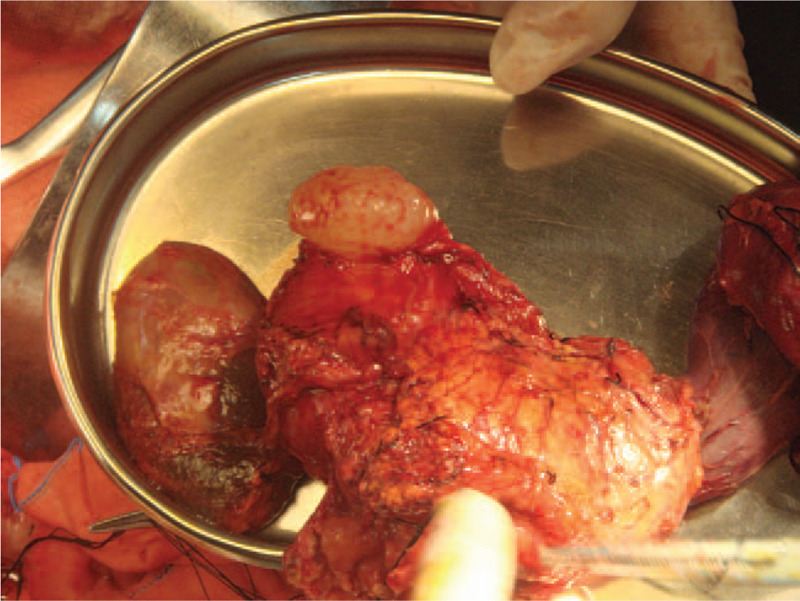
Photograph of the resected tumor. The cystic tumor appears to have originated from the head of the pancreas.

**Figure 7:**
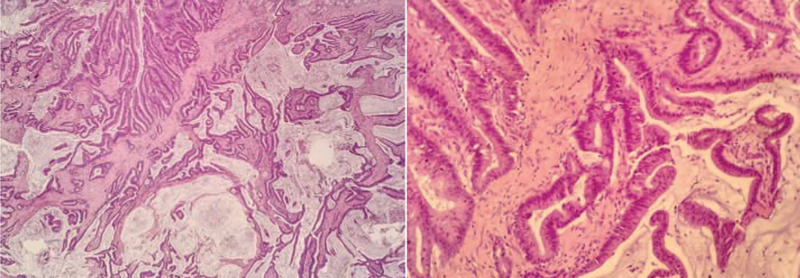
Histopathological findings. Postoperative pathology revealed a mucinous adenocarcinoma (hematoxylin and eosin staining; 40× and 200×).

Approximately 14 months after discharge from hospital the patient presented with complaints of dizziness and tiredness, which he had been experiencing for the past 6 months. Hemogram revealed iron-deficiency anemia (hemoglobin level: 65 g/L). Fecal occult blood test was positive. Tumor marker levels were as follows: CEA, 15.1 ng/mL; CA 19-9, 437.16 kU/L; and CA242, 146.04 kU/L. Magnetic resonance imaging of abdominal and pelvis revealed a cystic lesion with an irregular wall lying behind the head of the pancreas that had increased in size compared with the last abdominal CT (Fig. 3). Malignancy could not be ruled out on imaging. Upper gastrointestinal endoscopy revealed a jejunal tumor. Biopsy specimen sent for pathologic examination showed well-differentiated adenocarcinoma. Pancreatoduodenectomy was performed (Figs. 4 and 5). Intraoperatively, the cystic tumor appeared to arise from the head of the pancreas (Fig. 6). However, postoperative pathology of excised specimen revealed mucinous adenocarcinoma of the duodenum (Fig. 7). The cancer had invaded the capsule of the pancreas, but the pancreatic parenchyma was not involved. The surgical margin was negative, and there was no lymph node metastasis (0/3). TNM staging was T_4_N_0_M_0_.

At 10 days after the surgery, tumor marker levels were as follows: CEA, 3.83 ng/mL; CA 19-9, 48.13 kU/L; and CA242, 17.49 kU/L. At 27 days after surgery, tumor marker levels had returned to normal: CEA, 2.68 ng/mL; CA 19-9, 16.55 kU/L; and CA242, 5.61 kU/L. The patient refused chemotherapy, but has been attending yearly follow-up for the past 6 years. He has shown no signs of recurrence to date. Serum tumor marker levels and CT continue to be normal.

## 3 Discussion

Primary malignant duodenal tumors are very rare. They account for 20% to 25% of small intestine cancers, which themselves only constitute 5% of all alimentary tract tumors.^[6–9]^ According to the anatomic site, primary duodenal cancers can be divided into nonampullary duodenal cancers and periampullary duodenal cancers. Nonampullary duodenal cancers account for 0.5% of gastroenteric malignant tumors.^[10,11]^ Periampullary duodenal carcinoma is a type of periampullary carcinoma that derives from the end of the common bile duct, pancreatic duct, or Vater ampulla and involved duodenum.^[1–4]^ Periampullary duodenal carcinoma is less common.^[3,12]^ It accounts for 3% to 17% of periampullary cancers.^[5]^ In this paper, we present an extremely unusual case of periampullary duodenal cancer occurring 14 months after diagnosis of colon cancer.

For diagnosis of multiple primary malignant neoplasms (MPMN), the Warren and Gates criteria must be met, that is,

1. each malignancy must be histologically proven;
2. each tumor must be distinct from the other tumor(s); and
3. no tumor should be a metastasis from another.

If the time interval between the diagnoses of the 2 primary malignant neoplasms is <6 months, the MPMN is considered synchronous, and if it is >6 months, the MPMN is considered metachronous.^[13,14]^ By these criteria, our patient had metachronous MPMN.

Several authors have analyzed the incidence of a second primary malignancy in patients with previous diagnosis of colorectal cancer. After retrospective analysis of 55 cases, Li et al^[15]^ reported that gastric cancer was the most common cancer to be associated with colon cancer—accounting for 20% of the cases—followed by cancers of the esophagus, uterus, and lung. Only 1 patient had duodenal cancer; however, different from our patient, the duodenal cancer was diagnosed first and colorectal cancer was diagnosed >6 months later. Lee et al^[16]^ reported 35 metachronous MPMN patients with colorectal cancer as one of the primary neoplasms. Only 1 patient had small intestine cancer; the authors did not specify the site but, notably, the small intestine cancer was diagnosed before the colon cancer. In the study by Li et al,^[15]^ 48 of the 55 cases were metachronous, and only in 7 of the 48 cases were colorectal cancer diagnosed first. In the series reported by Lee et al,^[16]^ only in 4 of the 35 cases were colorectal cancer diagnosed first. Thus, in patients with metachronous cancers, it is obviously uncommon for colorectal cancer to be diagnosed first.

Vogt et al^[17]^ suggested that the risk of developing a second malignancy depends upon the site of the first malignancy. Weir et al^[18]^ found that second malignancies occur in 19.7% or 16.9% of patients with colon cancer and in 21% or 19.9% of patients with lung cancer, the percentages varying according to the criteria used (Surveillance Epidemiology and End Results project [SEER] or International Association of Cancer Registries and International Agency for Research on Cancer [IACR/IARC]).^[17,18]^ Amer et al^[19]^ reported a similar incidence of second cancers in patients with colon cancer, but a relatively lower incidence (5.6%) in patients with lung cancer. Second malignancies are reported in 1% of patients with liver cancers and in 16% of patients with bladder cancers.^[17]^

Broman et al^[20]^ reviewed the data of 12,064 colorectal cancer patients who developed a second primary gastrointestinal malignancy and reported that patients with colorectal cancer at any location had higher incidence of small intestine, bile duct, and other colorectal cancers; they also had lower incidence of liver and gallbladder cancer. The standardized incidence rate of small intestinal cancer was higher in patients with right colon cancer than in those with left colon or rectal cancer. Moreover, the expected incidence was fourfold higher than in the general population.^[20]^ Similarly, the risk of colon cancer was relatively higher in patients with small intestinal cancer.^[20]^ A possible explanation is that both small intestinal cancer and colorectal cancer are associated mutations of *KRAS*, *P53*, and *MMR* genes.^[20,21]^ However, in a population-based study in Denmark, Bojesen et al^[22]^ found no evidence of microsatellite instability in the 23 patients with Crohn disease who developed small bowel adenocarcinoma.

## 4 Conclusion

Clinicians should be aware that the incidence of small intestine cancer is higher in patients with colorectal cancer than in the general population; the risk is particularly high in patients with right colon cancer. Early detection and active surgical treatments can improve prognosis. Thus, long-term regular follow-up is necessary to detect new malignancies after colon cancer.

## Author contributions

**Conceptualization:** Tongjun Liu, Kai Zhang.

**Data curation:** Tao Li, Xinyu Wang, Nan Zhang, Wei Li.

**Resources:** Chen Chen, Xiaobin Song, Jiannan Li, Zeyun Zhao

**Supervision:** Tongjun Liu, Kai Zhang

**Writing – original draft:** Tao Li

**Writing – review & editing:** Tao Li, Tongjun Liu, Kai Zhang.
